# The Inhibitory Effect of Noscapine on the In Vitro Cathepsin G-Induced Collagen Expression in Equine Endometrium

**DOI:** 10.3390/life11101107

**Published:** 2021-10-19

**Authors:** Ana Amaral, Carina Fernandes, Anna Szóstek-Mioduchowska, Karolina Lukasik, Maria Rosa Rebordão, Pedro Pinto-Bravo, Dariusz Jan Skarzynski, Graça Ferreira-Dias

**Affiliations:** 1 CIISA—Centro de Investigação Interdisciplinar em Sanidade Animal, Departamento de Morfologia e Função, Faculdade de Medicina Veterinária, Universidade de Lisboa, 1300-477 Lisboa, Portugal; banasofiaamaral@fmv.ulisboa.pt (A.A.); fachica@hotmail.com (C.F.); milorebor-dao@gmail.com (M.R.R.); 2 Institute of Animal Reproduction and Food Research, Polish Academy of Science, 10-748 Olsztyn, Poland; a.szostek-mioduchowska@pan.olsztyn.pl (A.S.-M.); k.lukasik@pan.olsztyn.pl (K.L.); d.skarzynski@pan.olsztyn.pl (D.J.S.); 3 Polytechnic of Coimbra, Coimbra Agriculture School, Bencanta, 3045-601 Coimbra, Portugal; pbravo@esac.pt

**Keywords:** equine, endometrosis, fibrosis, collagen, cathepsin G, noscapine, inhibition

## Abstract

Cathepsin G (CAT) is a protease released by neutrophils when forming neutrophil extracellular traps that was already associated with inducing type I collagen (COL1) in equine endometrium in vitro. Endometrosis is a fibrotic condition mainly characterized by COL1 deposition in the equine endometrium. The objective was to evaluate if noscapine (an alkaloid for cough treatment with anti-neoplastic and anti-fibrotic properties) would reduce *COL1A2* transcription (evaluated by qPCR) and COL1 protein relative abundance (evaluated by western blot) induced by CAT in equine endometrial explants from follicular and mid-luteal phases treated for 24 or 48 h. The explants treated with CAT increased COL1 expression. Noscapine decreased *COL1A2* transcription at both estrous cycle phases, but COL1 relative protein only at the follicular phase, both induced by CAT. Additionally, the noscapine anti-fibrotic action was found to be more effective in the follicular phase. The CAT treatment caused more fibrosis at the longest period of treatment, while noscapine acted better at the shortest time of treatment. Our results showed that noscapine could act as an anti-fibrotic drug in equine endometrosis by inhibiting CAT in vitro. Noscapine offers a new promising therapeutic tool for treating fibrosis as a single non-selective agent to be considered in the future.

## 1. Introduction

Cathepsin G (CAT) is a protease released from neutrophils when they form neutrophil extracellular traps (NETs) in order to fight pathogens [1]. Neutrophil extracellular traps are composed of DNA filaments and enzymes such as elastase, myeloperoxidase, or CAT [1]. Although CAT’s main action is cleaving pathogen virulence factors [1,2], it is also associated with deleterious effects on the development of some diseases.

Recently, CAT was associated with the promotion of psoriasis in a mouse model [3]. Interestingly, a genetic variation that might increase CAT activity is related to cardiovascular, neuromuscular, and osteomyelitis diseases [4,5,6]. In other mouse models, CAT was linked to fibrotic conditions, such as chronic obstructive pulmonary disease (COPD) [7] and kidney fibrosis [8]. In humans, some reports have also associated CAT to the course of lung cystic fibrosis [9,10], COPD [10,11], and Dupuytren’s hand contracture [12]. 

Recently, our team investigated the effect of some enzymes released from NETs on collagen type I production (COL1) by equine endometrial explants [13,14,15,16,17]. The treatment of mare’s endometrial explants with NETs’ components revealed an increased COL1 content. The COL1 increase is the hallmark of a chronic degenerative fibrotic disease in the mare endometrium called endometrosis. Thus, these findings suggested that elastase, CAT, and myeloperoxidase might be involved in the development of endometrosis [13,14,15,16,17].

After semen arrival at the equine uterine lumen, a physiological breeding-induced endometritis with increased pro-inflammatory cytokines is mounted to remove the excess of spermatozoa, microorganisms, or debris [18,19,20,21]. This fast neutrophil influx to the uterus must be solved until 24 h post-breeding. Otherwise, mares will be predisposed to develop a chronic endometritis responsible for continuous neutrophil influx to the uterine lumen [22,23]. In turn, the chronic exposure of endometrium to NET components might contribute to endometrosis development [13,14,15,16,17,24]. Endometrosis is one of the main causes of mare infertility since the normal endometrial parenchyma is replaced, and increased deposition of collagen in the lamina propria occurs [25]. Therefore, the endometrial glands gather in nests surrounded by COL fibers, compromising glandular function and histotrophic secretion, and ultimately impairing early embryonic maintenance [25,26,27]. Despite some endometrosis treatments advocated for over the last decades (mechanical curettage, dimethyl sulfoxide, kerosene, or stem cells), they were revealed to be unsuccessful [28,29,30,31,32]. We have demonstrated, in equine endometrial explants, that it is possible to inhibit in vitro the pro-fibrotic effect of elastase [14,15], CAT [16], and myeloperoxidase [17], using selective inhibitors of these enzymes. In fact, cathepsin G Inhibitor I (β-keto-phosphonic acid) is a selective CAT inhibitor that reduced CAT-induced COL1 in explants of mares’ endometria [16]. These findings are a newly promising approach to treating equine endometrosis. However, inhibiting the enzymes found in NETs in a selective way may reveal itself to be not highly effective because of the multifactorial etiology of equine endometrial fibrosis. 

Noscapine (NOSC) is an alkaloid extracted from poppy that is used to treat cough [33,34] and cancer [34,35]. In fact, NOSC has shown low toxicity [36] and adequate pharmacokinetics in mice as an anti-cancer drug [37] and drives distinct apoptotic pathways in different cell lines of cancer models [38,39,40,41]. Ke et al. [36] reported that mice treated orally with NOSC presented little to no toxicity in the heart, kidney, liver, spleen, or bone marrow at tumor-suppressive doses. Although the small intestine has shown mild nonspecific toxicity, no apoptosis or disease were found in this organ, suggesting that the healthy tissues are more resistant to apoptotic effects of NOSC than neoplasic tissues [36]. However, some reports described the occurrence of toxicity in experimental animals and humans after NOSC administration but at higher doses [42,43]. After 24 h of IV injection of NOSC in mice, 85% of it was excreted, but the remaining 25% could lead to toxicity effects if NOSC was administered daily [42]. Moreover, 20% of terminal human cancer patients showed side effects, as mild sedation, and abdominal discomfort, after higher doses of NOSC (3000 mg daily) [43]. Interestingly, NOSC delivered in nanoparticles in vitro allowed the administration of a higher NOSC concentration, while the toxicity effects were kept to a minimum [44].

Noscapine was also tested as an anti-fibrotic drug in vivo in mice and in vitro in pulmonary fibroblasts, showing a new anti-fibrotic action [45]. In addition, in triple negative breast cancer in mice, NOSC reduced the fibrosis associated with the tumor [46]. We have recently investigated the anti-fibrotic in vitro effect of NOSC on equine endometrial explants challenged with elastase by reducing COL1 production [47]. This new finding showed that it is possible to inhibit elastase in a non-selective way using NOSC in vitro, opening new therapeutic strategies to fight equine endometrosis.

Therefore, we aimed to investigate if NOSC acted as an anti-fibrotic drug in equine endometrial explants when exposed to CAT pro-fibrotic action. Specifically, in this study, the putative in vitro inhibitory action of NOSC on CAT-induced collagen type I alpha 2 chain (*COL1A2*) mRNA and COL1 protein relative abundance on equine endometrial explants from different estrous cycle phases and times of treatment was investigated.

## 2. Materials and Methods

### 2.1. Mares

Uteri and jugular venous blood were collected from healthy mares euthanized at an abattoir in Poland according to European legislation (European Food Safety Authority, AHAW/04-027). As described previously [13,48], the estrous cycle phase determination was based on the observation of uterine and ovarian structures and later confirmed by plasma progesterone (P4) concentration evaluation. Briefly, the mares were considered in the follicular phase (FP) if presented a follicle >35 mm diameter and P4 concentration <1 ng/mL. If mares presented a well-developed corpus luteum and P4 plasma concentration, >6 ng/mL were considered in mid-luteal phase (MLP). Only uteri that presented no signs of endometritis (increased abnormal mucus production, altered coloration of endometrium surface, and presence of bacteria or neutrophils) [13,24,49] and classified as category IIA and IIB [50], corresponding to mild to moderate histopathological alterations of endometrosis [50], were considered for this study. Immediately after retrieval, the uteri from FP (*n* = 8) and MLP (*n* = 7) were transported on ice immersed in cold Dulbecco’s modified Eagle’s medium (DMEM) F-12 Ham medium (D/F medium; 1:1 (*v*/*v*); D-2960; Sigma-Aldrich, St Louis, MO, USA), supplemented with 100 IU/mL penicillin (P3032; Sigma-Aldrich, St Louis, MO, USA), 100 μg/mL streptomycin (S9137; Sigma-Aldrich, St Louis, MO, USA), and 2 μg/mL amphotericin (A2942; Sigma-Aldrich, Burlington, MA, USA) to the laboratory.

### 2.2. In Vitro Culture of Mare Endometrial Explants

Collection and preparation of endometrial explants were performed as described previously [15]. The explants were pre-incubated for 1 h, at 38 °C and 5% CO_2_, in a humidified atmosphere chamber (Biosafe Eco-Integra Biosciences, Chur, Switzerland) in a DMEM culture medium supplemented with 2 µg/mL amphotericin (A2942; Sigma-Aldrich, St Louis, MO, USA), 100 IU/mL penicillin (P3032; Sigma-Aldrich), 100 µg/mL streptomycin (S9137; Sigma-Aldrich, St Louis, MO, USA), and 0.1% (*w*/*v*) bovine serum albumin (BSA; 735078; Roche Diagnostics, Mannheim, Germany). Afterwards, the culture medium was replaced, and the explants further treated for 24 or 48 h, as follows: (i) vehicle (negative control)—culture medium; (ii) CAT (0.1 µg/mL or 1 µg/mL; A6942, Applichem GmbH, Germany); (iii) noscapine hydrochloride hydrate (NOSC; 45 µg/mL; N9007; Merck, Darmstadt, Germany); (iv) CAT (0.1 µg/mL or 1 µg/mL) + NOSC (45 µg/mL). The individual treatments were carried out in quadruplicate. The NOSC treatment was performed just after culture medium replacement and CAT added 1 h after NOSC treatment. The CAT 0.1 and 1 µg/mL concentrations were chosen because they had already proven to induce COL1 expression in equine endometrial explants [13,16]. The concentration of NOSC was previously validated by a pre-trial assay (data not shown) evaluating the concentrations of 0.45, 4.5, 45, 450, and 4500 µg/mL used on previous studies [40,45]. Furthermore, the concentration of NOSC that was able to inhibit COL1 elastase-induced in equine endometrial explants was 45 µg/mL [47]. After the treatment period, the explants and culture media were collected in RNAlater® (R901, Sigma-Aldrich, St Louis, MO, USA) or in 0.3 M ethylenediaminetetraacetic acid (E5134, Sigma-Aldrich, St Louis, MO, USA) + 1% aspirin (A2093, Sigma-Aldrich, St Louis, MO, USA) solution, respectively, and stored at −80 °C. 

### 2.3. Viability of Equine Endometrial Explants

The viability of equine endometrial explants was assessed by lactate dehydrogenase (LHD) activity [15]. 

Lactate dehydrogenase is released to the extracellular space if the cell membrane is damaged. Hence, the LDH activity was measured in conditioned culture media and in explants incubated for 1, 24, and 48 h, using a colorimetric assay kit (ab 102526, Abcam, Cambridge, UK) according to the manufacturer’s protocol and as optimized by Amaral et al. [15]. Afterwards, the quotient of the intracellular LDH activity and the total activity (extracellular plus intracellular LDH) indicated the explant viability [51].

### 2.4. Determination of COL1A2 mRNA Transcription by Real-Time Polymerase Chain Reaction (qPCR) 

The mRNA was extracted from explants using TRI Reagent^®^ (T9424; Sigma-Aldrich, St Louis, MO, USA.) according to the manufacturer’s instructions. The mRNA quantification was assessed using a Nanodrop system (ND 200C; Fisher Scientific, Hamton, PA, USA), and mRNA quality was evaluated by visualization of 28S and 18S rRNA bands after electrophoresis of a 1.5% red staining agarose gel (41,003; Biotium, Hayward, CA, USA). The synthesis of cDNA was performed using M-MLV reverse transcriptase enzyme (M5313; Promega, Madison, WI, USA) from 1 μg of total RNA in a 20 μL reaction volume using oligo (dT) primer (C1101; Promega, Madison, WI, USA).

The validation of reference ribosomal protein L32 (*RPL32*) and target *COL1A2* genes was performed as described by Amaral et al. [15]. The equine-specific primer sequences are listed in Table 1. The qPCR reactions of both genes were run in duplicate in the StepOnePlus™ Real-Time PCR System (Applied Biosystems, Warrington, UK) in a 96-well plate (4306737; Applied Biosystems, Warrington, UK) and product specificity was analyzed, as previously described [15,52].

### 2.5. Quantification of COL1 Protein Relative Abundance by Western Blot

The preparation of samples for protein extraction (RIPA buffer supplemented with protease inhibitor), as well as protein quantification (Bradford reagent), was described previously [15]. The protein extract (30 µg) in 2x Laemmli Loading Buffer and DTT was loaded on 8% acrylamide gel (MB04501; Nzytech, Lisbon, Portugal) incorporated with 0.5% (*v*/*v*) 2,2,2-trichloroethanol (808610; Merck, Darmstadt, Germany). After, the samples were run and transferred to a nitrocellulose membrane (GE10600001, GE Healthcare, Chicago, IL, USA) as described before [15]. To accomplish band normalization and comparison between gels, a standard endometrial sample (30 µg) was loaded in a single lane. To perform the non-staining total protein loading control, the membranes were exposed for 1 min to UV light (ChemiDoc XRS + System, Bio-Rad, Hercules, CA, USA) to obtain the normalization image. The COL1 primary antibody (1:1000 diluted; RRID: AB_2891017, 20121, Novotec, Lyon, France) was incubated overnight at 4 °C and previously validated to equine endometrium by Rebordão et al. [13]. Then, the membranes were incubated for 1.5 h at room temperature with the secondary antibody horseradish peroxidase (HRP)-conjugated anti-rabbit (1:20,000; RRID: AB_2617138; P0448, DakoCytomation, Carpinteria, CA, USA). The bands of COL1 were detected using luminol-enhanced chemiluminescence (Super Signal West Pico, 34077; Thermo Scientific, Waltham, MA, USA) and then analyzed by Image Lab 6.0 (Bio-Rad, Hercules, CA, USA) software using a multichannel protocol, detecting the total protein lanes in the stain-free total protein membrane image and COL1 bands on the chemiluminescence image [55]. The amount of COL1 protein was calculated by a factor of normalization to adjust the variability of the loaded protein [55].

### 2.6. Statistical Analysis

The LDH results were evaluated by one-way analysis of variance (ANOVA) followed by a Tukey’s multiple comparisons test (GraphPAD PRISM, Version 6.00, 253 GraphPad Software, San Diego, CA, USA). The viability data are displayed as mean ± SEM and considered significant at *p* < 0.05. The test of Kolmogorov–Smirnov of Proc Univariate of SAS v. 9.4 (SAS Institute Inc., Cary, NC, USA) and visual evaluation were used to assess data normality. Since the *COL1A2* transcription and COL1 protein relative abundance variables did not show a normal distribution, data were converted using the square root.

In order to assess the response of *COL1A2* mRNA and COL1 protein relative abundance to the different treatments performed (24 combinations of concentration of CAT, effect of NOSC, estrous cycle phase, and time of treatment), data were analyzed using PROC GLM (SAS v. 9.4; SAS Institute Inc., Cary, NC, USA) in two steps. First, we used a model where the response variables were affected by the various treatments considered, in a total of 24 treatment combinations. 

In a second analysis, the factorial nature of the factors included in our study was considered. The main effects included were pro-fibrotic factor (CAT: 0, 0.1 and 1.0 μg/mL), anti-fibrotic factor (NOSC: 0 and 0.45 μg/mL), estrous cycle phase (FP and MLP), and time of treatment (24 and 48 h). In addition to the main effects, all possible two-way, three-way, and four-way interactions were considered in the statistical analyses. 

Subsequently, the least square means for various treatment combinations were compared with the PDIFF of PROC GLM, and the results were considered significant at *p* < 0.05. The least squares means ± SEM were then back-transformed and presented graphically with GraphPAD PRISM (Version 6.00, 253 GraphPad Software, San Diego, CA, USA). 

## 3. Results

### 3.1. Evaluation of Equine Endometrial Explant Viability

The results of LDH activity for 1, 24, and 48 h of incubated endometrial explants are presented in Table 2. There was a statistical difference between 1 h of incubation and the other two times of incubation (24 or 48 h) (*p* < 0.05). The results were independent of estrous cycle phase.

### 3.2. The Isolated Effect of CAT, NOSC, Time of Treatment, and Estrous Cycle Phase and Their Interaction Combinations

In Table 3 are listed the isolated effects of CAT, NOSC, time of treatment, and estrous cycle phase for both *COL1A2* mRNA transcription and COL1 protein abundance. Table 3 also shows the significance for all the interactions between factors for both *COL1A2* mRNA transcription and COL1 protein abundance. 

### 3.3. The Noscapine Inhibition of COL1 Induced by CAT Is Independent of Estrous Cycle Phase and Time of Treatment

**Figure 1:**
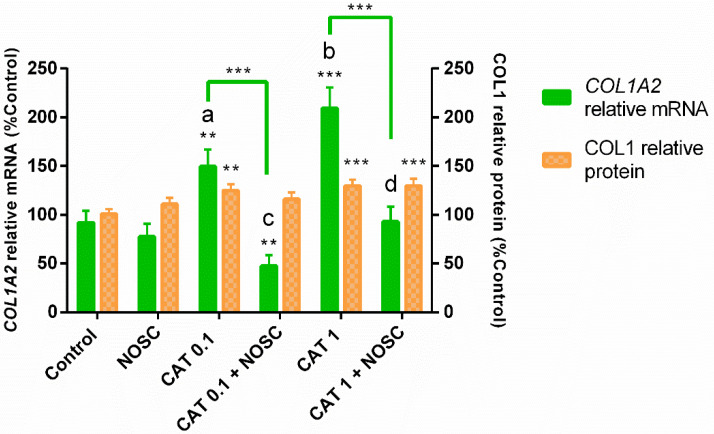
Inhibition of cathepsin G (0.1 or 1 µg/mL) by noscapine (NOSC; 45 µg/mL) on relative collagen type I alpha 2 chain (*COL1A2*) mRNA transcription and collagen type I (COL1) protein relative abundance in mare endometrial explants, regardless of estrous cycle phase and time of treatment. Results were considered significant at *p* < 0.05 and are displayed as least square means ± SEM. Different superscript letters indicate significant differences between CAT concentrations (a, b: CAT 0.1 µg/mL ≠ CAT 1 µg/mL, *p* < 0.05; c, d: CAT 0.1 µg/mL + NOSC ≠ CAT 1 µg/mL + NOSC, *p* < 0.05). Asterisks alone represent significant differences relative to the respective control and asterisks above the connecting lines indicate significant differences between treatments (** *p* < 0.01; *** *p* < 0.001).

Both concentrations of CAT were capable of increasing *COL1A2* mRNA transcription and COL1 protein relative abundance (CAT 0.1 µg/mL: *p* < 0.01; CAT 1 µg/mL: *p* < 0.001; Figure 1). However, CAT 1 µg/mL increased *COL1A2* mRNA transcription the most (*p* < 0.05; Figure 1). The NOSC inhibitory effect was only detected in *COL1A2* mRNA transcription induced by both concentrations of CAT (*p* < 0.001; Figure 1). The combination of CAT 0.1 µg/mL + NOSC also reduced *COL1A2* mRNA transcription when compared to control group (*p* < 0.01; Figure 1). Nevertheless, CAT 0.1 µg/mL + NOSC treatment reduced the most *COL1A2* mRNA transcripts when compared to CAT 1 µg/mL + NOSC treatment (*p* < 0.05; Figure 1). Western blot analysis revealed that NOSC did not reduce COL1 induced by either CAT concentrations, and that CAT 1 µg/mL + NOSC treatment remained increased when compared to the non-treated explants (*p* < 0.001; Figure 1). 

Regarding *COL1A2* mRNA transcription, the NOSC treatment differed from CAT 0.1 and 1 µg/mL treatments, and the differences are shown in Supplementary Table S1.

### 3.4. Noscapine Inhibition of CAT-Induced COL1 Expression Is Different Depending on the Estrous Cycle Phase and Treatment Time

**Figure 2:**
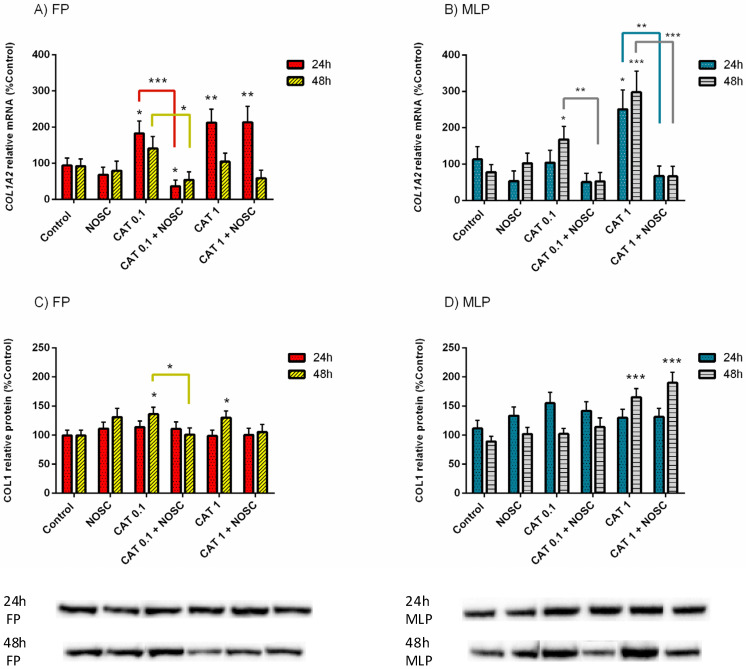
Effect of cathepsin G (CAT; 0.1 or 1 µg/mL), noscapine (NOSC; 45 µg/mL), or CAT (0.1 or 1 µg/mL) + NOSC (45 µg/mL) treatments in explants of mare endometrium from follicular phase (FP) or mid-luteal phase (MLP) for 24 or 48 h on relative collagen type I alpha 2 chain (*COL1A2*) mRNA transcription (**A**,**B**) and collagen type I (COL1) protein relative abundance (**C**,**D**). Results were considered significant at *p* < 0.05 and shown as least square means ± SEM. Asterisks alone represent significant differences relative to the respective control and asterisks above connecting lines indicate significant differences of CAT + NOSC treatment relative to the respective CAT-treated group (* *p* < 0.05; ** *p* < 0.01; *** *p* < 0.001).

In FP, the treatment with CAT 0.1 µg/mL increased *COL1A2* mRNA transcription at 24 h (*p* < 0.05; Figure 2A) and COL1 relative protein abundance at 48 h (*p* < 0.05; Figure 2C), with respect to the respective control groups. However, the addition of NOSC reduced both *COL1A2* mRNA transcription (24 h, *p* < 0.001; Figure 2A) and COL1 protein relative abundance (48 h, *p* < 0.05; Figure 2C), regarding CAT 0.1 µg/mL treated groups. Also in FP, at 48 h, the combined treatment of CAT 0.1 µg/mL + NOSC decreased *COL1A2* mRNA transcription when compared to CAT 0.1 µg/mL treated group (*p* < 0.05; Figure 2A), which was not increased compared to control. Cathepsin G 1 µg/mL treatment upregulated *COL1A2* mRNA transcripts at 24 h (*p* < 0.01; Figure 2A) and COL1 relative protein abundance at 48 h (*p* < 0.05; Figure 2C), respective to the control groups, both in FP. Noscapine did not inhibit these effects of CAT 1 µg/mL. Furthermore, in the CAT 1 µg/mL + NOSC treated group, the *COL1A2* transcription remained augmented compared to control (*p* < 0.01; Figure 2A).

In MLP, CAT 0.1 µg/mL increased *COL1A2* mRNA transcription at 48 h compared to the control group (*p* < 0.05; Figure 2B), but the addition of NOSC reduced this effect (*p* < 0.01; Figure 2B). In addition, in MLP, CAT 1 µg/mL treatment was capable of increasing *COL1A2* mRNA transcripts when compared to the control at both 24 h (*p* < 0.05; Figure 2B) and 48 h (*p* < 0.001; Figure 2B). Nevertheless, explant treatment with CAT 1 µg/mL + NOSC diminished *COL1A2* mRNA transcription in MLP at both 24 h (*p* < 0.01; Figure 2B) and 48 h (*p* < 0.001; Figure 2B) when compared to the respective CAT 1 µg/mL treated groups. The COL1 protein abundance augmented with CAT 1 µg/mL and CAT 1 µg/mL + NOSC treatments, relative to the respective control groups, in MLP at 48 h (*p* < 0.001; Figure 2D).

The differences between NOSC treatment and the other performed treatments are shown in supplementary Table S2. The differences found for the same treatments between 24 and 48 h of incubation time, within each estrous cycle phase, are listed in Supplementary Table S3. In Supplementary Table S4 are presented the differences found for the same treatments, between FP and MLP, within each treatment time. In Supplementary Table S5 are listed the means and SEM of *COL1A2* transcription and COL1 protein abundance for all the treatments performed in equine endometrial explants from FP or MLP treated for 24 or 48 h. In Supplementary Tables S6 and S7 are shown the significance levels (*p* values) between all the performed treatments in equine endometrial explants from FP or MLP treated for 24 or 48 h in the analyses of relative transcript *COL1A2* gene and COL1 protein abundance, respectively. 

### 3.5. Noscapine Inhibition on CAT-Induced COL1 Expression Is Dependent of Treatment Time

**Figure 3:**
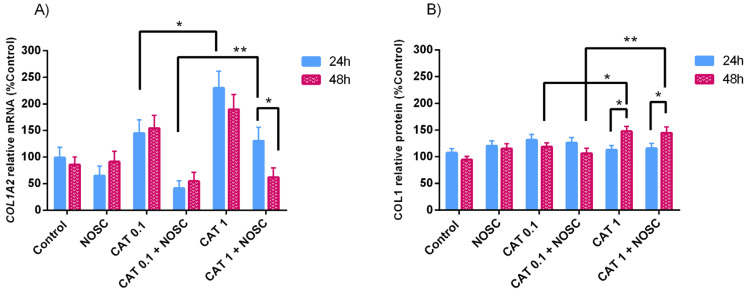
Effect of cathepsin G (CAT; 0.1 or 1 μg/mL), noscapine (NOSC; 45 μg/mL), or CAT (0.1 or 1 μg/mL) + NOSC (45 μg/mL) treatments on relative collagen type I alpha 2 chain (*COL1A2*) mRNA transcription (**A**) and collagen type I (COL1) protein relative abundance (**B**) in equine endometrial explants treated for 24 or 48 h, regardless of estrous cycle phase. Results are shown as least square means ± SEM and considered significant at *p* < 0.05. Asterisks above connecting lines indicate significant differences of the same treatment between time of treatment or differences between different concentrations of CAT at the same time of treatment (* *p* < 0.05; ** *p* < 0.01).

Cathepsin G 1 μg/mL increased *COL1A2* mRNA transcription at 24 h and COL1 protein relative abundance at 48 h to a higher extent than CAT 0.1 μg/mL did (*p* < 0.05; Figure 3A,B). In contrast, CAT 0.1 μg/mL + NOSC downregulated *COL1A2* mRNA transcripts at 24 h and COL1 protein relative abundance at 48 h more than CAT 1 μg/mL + NOSC, (*p* < 0.01; Figure 3A,B). At 48 h, the treatment with CAT 1 μg/mL + NOSC decreased the most *COL1A2* mRNA transcription in comparison with 24 h incubation time (*p* < 0.05; Figure 3A). The treatment with CAT 1 μg/mL increased the most COL1 protein relative abundance at 48 h relative to 24 h. Although, the combined treatment of CAT 1 μg/mL + NOSC provoked a steeper decrease of COL1 protein relative abundance at 24 h than at 48 h (*p* < 0.05; Figure 3B). 

In Supplementary Table S8 are listed the means and SEM of *COL1A2* transcription and COL1 protein abundance for all the treatment performed in equine endometrial explants treated for 24 or 48 h, independent of estrous cycle phase. In Supplementary Tables S9 and S10 are shown the significance levels (*p* values) between all the performed treatments in equine endometrial explants treated for 24 or 48 h in the analyses of relative transcript *COL1A2* gene and COL1 protein abundance, respectively. 

### 3.6. The Noscapine Inhibition on CAT-Induced COL1 Expression Is Dependent on Estrous Cycle Phase

**Figure 4:**
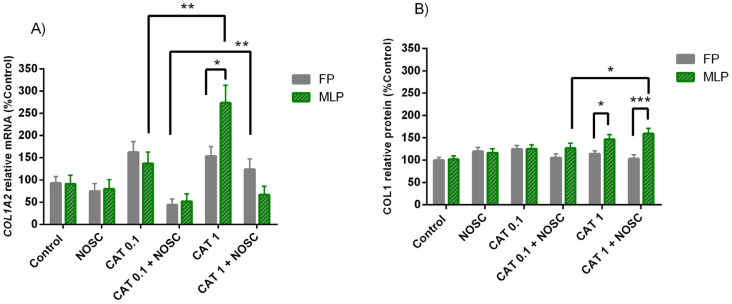
Effect of cathepsin G (CAT; 0.1 or 1 μg/mL), noscapine (NOSC; 45 μg/mL), or CAT (0.1 or 1 μg/mL) + NOSC (45 μg/mL) treatments on relative collagen type I alpha 2 chain (*COL1A2*) mRNA transcription (**A**) and collagen type I (COL1) protein relative abundance (**B**) in explants of mare endometrium from follicular (FP) or mid-luteal (MLP) phases, regardless of treatment time. Results are shown at least square means ± SEM and considered significant at *p* < 0.05. Asterisks above connecting lines indicate significant differences of the same treatment between estrous cycle phase or differences between different concentrations of CAT at the same estrous cycle phase (* *p* < 0.05; ** *p* < 0.01; *** *p* < 0.001).

In MLP, CAT 1 μg/mL increased the most *COL1A2* mRNA transcription compared to CAT 0.1 μg/mL (*p* < 0.01; Figure 4A). The combination of CAT 0.1 μg/mL + NOSC diminished *COL1A2* mRNA transcripts in FP (*p* < 0.01; Figure 4A) and COL1 relative protein abundance in MLP (*p* < 0.05; Figure 4B) more than CAT 1 μg/mL + NOSC. In MLP, CAT 1 μg/mL elevated both *COL1A2* mRNA transcription and protein relative abundance of COL1 more than in FP (*p* < 0.05; Figure 4A,B), whereas CAT 1 μg/mL + NOSC decreased COL1 protein relative abundance in FP more than MLP (*p* < 0.001; Figure 4B).

In Supplementary Table S11 are listed the means and SEM of *COL1A2* transcription and COL1 protein abundance for all the treatments performed in equine endometrial explants from FP or MLP, independent of treatment time. In Supplementary Tables S12 and S13 are shown the significance levels (*p* values) between all the performed treatments in equine endometrial explants from FP or MLP in the analyses of relative transcript *COL1A2* gene and COL1 protein abundance, respectively. 

## 4. Discussion

Such as in other organs, equine endometrial fibrosis has many triggering factors that may contribute to endometrosis by distinct pathways. Not only TGFβ1 and other cytokines (IL-1α, IL-1β, IL-6) [56,57], but also prostaglandins [14,24,49,58] were linked to equine endometrosis. The release to the extracellular environment of some enzymes when neutrophils form NETs, such as elastase, myeloperoxidase, and CAT, has also proven to be capable of increasing COL1 expression in vitro in equine endometrium, despite their beneficial antimicrobial action [13].

**Figure 5:**
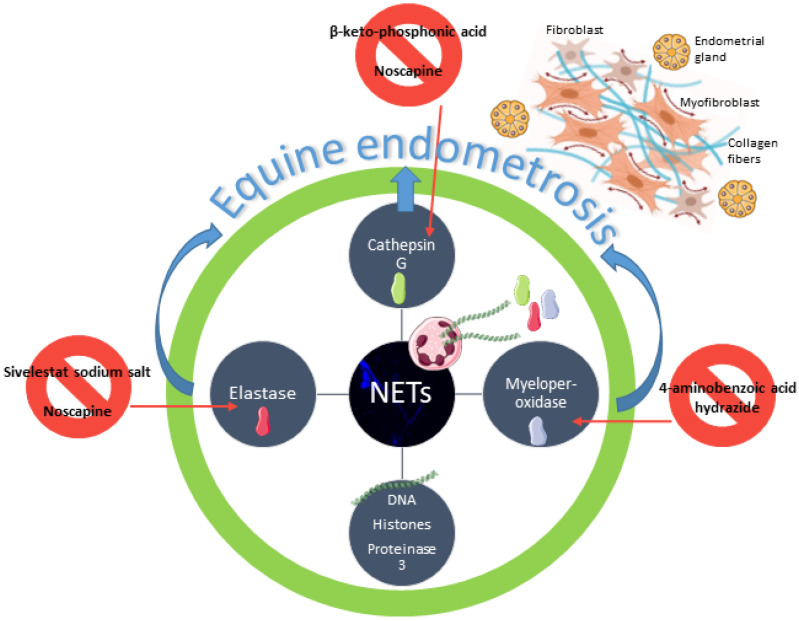
Schematic representation of neutrophil extracellular traps (NETs) putative involvement in equine endometrosis establishment. Endometrosis is characterized by fibroblast differentiation into myofibroblasts and collagen deposition around the endometrial glands [25,26,27]. A continuous influx of neutrophils to the endometrium leads to excessive formation of NETs and long standing of NETs components, such as DNA, histones, proteinase 3, elastase, cathepsin G, and myeloperoxidase [1]. Elastase, cathepsin G, and myeloperoxidase are capable of inducing collagen deposition in the equine endometrium [13]. Sivelestat sodium salt, β-keto-phosphonic acid, and 4-aminobenzoic acid hydrazide are selective inhibitors of elastase, cathepsin G, and myeloperoxidase that reduced collagen deposition in equine endometrium explants [15,16,17]. Noscapine, an alkaloid used to treat cough, cancer, and fibrosis, also reduced collagen in vitro deposition in equine endometrium, induced by elastase [47] and cathepsin G.

Due to the challenging of finding an effective equine endometrosis treatment, we have been focusing our latest in vitro studies in new possible ways to decrease collagen deposition in equine endometrium. We have already demonstrated that elastase, myeloperoxidase, or CAT were inhibited by their selective inhibitors and thus capable of decreasing COL1 expression in equine endometrial explants [14,15,16,17] (Figure 5). These recent findings may contribute to the advance of a new prophylactic or therapeutic method for endometrosis, based on this evidence of in vitro reduction of COL1 in mare endometrium, induced by enzymes found in NETs. Cathepsin G Inhibitor I (β-keto-phosphonic acid) is the selective CAT inhibitor successfully tested in vitro in equine endometrium that reduced COL1 induced by CAT [16] (Figure 5).

Not underestimating these results, we have decided to investigate the effect of a non-selective inhibitor of some enzymes present in NETs. In equine endometrial explants treated with elastase, NOSC reduced the elastase pro-fibrotic effect by reducing COL1 expression [47] (Figure 5). The next step was to evaluate if NOSC was also able to inhibit COL1 induced by CAT in mare endometrial explants. In the present study, both concentrations of CAT (0.1 and 1 µg/mL) induced COL1 expression. However, NOSC only inhibited *COL1A2* mRNA transcription, regardless of estrous cycle phase and time of treatment.

Comparing the response of endometrial explants from FP and MLP, both increased COL1 expression when treated with the two CAT concentrations tested, but the inhibition by NOSC only decreased COL1 relative protein abundance in FP. In MLP, the NOSC treatment did not reduce the pro-fibrotic action of CAT 1 µg/mL, which persisted. Moreover, CAT 1 µg/mL had a higher pro-fibrotic effect in MLP than FP. The lowest concentration of CAT seems to be better controlled by NOSC than the highest concentration used. Additionally, the NOSC anti-fibrotic action was found to be more effective in FP.

In the FP, estrogens increase uterine blood flow in the mare, thus stimulating the local immune response as well [59]. This fact may contribute to the reduction of endometritis/endometrosis chronicity, explaining how the COL1 protein relative abundance was inhibited by NOSC only in FP endometrial explants, physiologically primed by estrogens. Conversely, in MLP, under progesterone influence, the immune response subsides [59], causing a predisposition to the persistence of chronic stimuli in the uterus. This might explain why MLP explants were more reactive to CAT 1 µg/mL pro-fibrotic effects than FP explants and why NOSC did not inhibit COL1 protein abundance induced by CAT in MLP explants.

Additionally, CAT 1 µg/mL, when compared to CAT 0.1 µg/mL, showed a higher increase of COL1 protein relative abundance at 48 h. However, the combined treatment of CAT 1 µg/mL + NOSC was more effective in reducing it at 24 h than after a 48 h incubation. The highest concentration of CAT was more prone to induce COL deposition at the longest period of treatment, while NOSC treatment as an anti-fibrotic agent was more efficient at the shortest time of treatment. Thus, the highest concentration of CAT was responsible for the greatest pro-fibrotic effect. 

Despite the NOSC concentration (45 µg/mL) being the same as that one successfully used in elastase treatment of mare endometrial explants [47], it may be suggested that this concentration is not enough to inhibit the CAT pro-fibrotic effect. Few inhibitory effects were detected in COL1 protein relative abundance, especially in explants challenged by the highest concentration of CAT. However, NOSC successfully inhibited *COL1A2* mRNA transcripts in CAT-treated endometrial tissue at both estrous cycle phases. Further studies must be considered using a higher concentration of NOSC to evaluate the inhibition of COL1 protein induced by CAT. Furthermore, the dose trial assay for β-keto-phosphonic acid determined that this CAT selective inhibitor had to be administered to the explant culture not only at the beginning of the treatment time but also at 24 h for those explants treated for 48 h [16]. The second administration of NOSC after the 24 h treatment could have been sufficient to overcome the lack of inhibition observed at 48 h of treatment. It might be suggested that an increase in NOSC concentration, both at 24 and at 48 h, would be needed to reach the desirable inhibitory effect of fibrosis.

However, these new findings contribute to a better understanding of the use of NOSC in equine endometrosis treatment or prophylaxis. Since noscapine has been administered to in vivo cancer models [39] and to treat cough in humans since 1930 [33,34], it facilitates its in vivo use compared to other inhibitors not tested in vivo. Noscapine has been revealing many applications in the last years. It was recently studied to be used in COVID-19 treatment, given that it acts as a protease inhibitor of the virus [60]. It was also reported to act as an anti-inflammatory drug by acting on cytokine regulation [61] and by impairing mediators of inflammation [62]. Additionally, in many cell lines or mice models, NOSC was effective as an anti-neoplasic agent [34,35,63]. By binding to tubulin, NOSC modifies its conformation and attenuates microtubules. This way, microtubules stay longer in a paused mode, leading to a block in mitosis at prometaphase, thus inducing apoptosis of neoplasic cells [38,64,65]. However, NOSC does not cause apoptosis of benign cells because neoplasic cells lack normal mitotic spindle assembly checkpoint [38,66]. Endometrosis is characterized by the differentiation of myofibroblasts, which are the main source of collagen and other extracellular matrix components [26,58]. Once NOSC could inhibit myofibroblast differentiation by binding to microtubules [67], this might be a possible anti-fibrotic mechanism of action of this drug in mare endometrium. 

One of the pathways that seems to be involved in anti-neoplasic action of NOSC is the inhibition of nuclear factor kappa-light-chain-enhancer of activated B cells (NF-kB) pathway [68,69,70]. Besides the role of this pathway in immune response, a dysregulation of NF-kB pathway also leads to inflammatory and neoplasic disorders. Moreover, Dong and Ma [71] demonstrated that the NF-kB signaling pathway mediates the activation of pro-fibrotic genes in fibroblastic pulmonary cells, triggering fibrosis progression of the lungs. Interestingly, NOSC inhibited the NF-kB pathway in human leukemia and myeloma cells [68], mice models of breast cancer [69], and ovarian cancer cells [72]. Also in the mare, the NF-κB pathway may be involved in the progression of endometrosis, specifically in the FP, suggesting a hormone-dependent manner for activation of fibrogenesis by these signaling proteins [73]. Similarly, in our present work, since COL1 protein relative abundance in endometrial explants was inhibited by NOSC only in the FP, its anti-fibrotic action might be mediated by the NF-kB pathway. However, further studies must be pursued to evaluate this hypothesis.

Regarding the effect of NOSC as an anti-fibrotic agent, fewer studies are found. Kach et al. [45] reported that NOSC impaired the TGFβ-induced stress fiber, without influencing the content of the microtubule, in cultured human lung fibroblasts. The same authors also concluded that NOSC exerted its anti-fibrotic effects through prostaglandin E2 receptor (EP2), which, in turn, activates protein kinase A (PKA) [45]. We have already demonstrated that EP2 mediates the anti-fibrotic effect of prostaglandin (PG)E_2_ in equine endometrial explants that were treated with elastase, CAT, or myeloperoxidase [49]. While CAT increased COL1 expression in equine endometrial explants, it also decreased PGE_2_ or EP2 transcripts [49]. Likewise, the use of sivelestat sodium salt as a selective elastase inhibitor augmented PGE_2_ secretion in vitro by equine endometrium, suggesting that PGE_2_ may have an anti-fibrotic effect in equine endometrium [14]. Once tumor collagen content decreased the penetration of anti-neoplasic drugs, the effect of oral low dose administration of NOSC, as a solid tumor anti-fibrotic agent, was investigated in mice [46]. These authors observed that NOSC reduced COL1 content in triple-negative breast cancer solid tumors, showing a NOSC anti-fibrotic effect [46]. Recently, Cabezas et al. [32] suggested the use of PGE_2_ as an anti-fibrotic agent, since the in vitro use of PGE_2_ (mediated by EP2) preconditioning equine adipose mesenchymal stem cells improved their immunomodulatory competence. Notwithstanding the described putative mechanisms of action, further studies must be carried out to understand how NOSC works as an anti-fibrotic drug in equine endometrium.

## 5. Conclusions

By inhibiting more than one triggering factor of endometrosis, the use of NOSC simplifies the therapeutic approach by administering a single agent and offers a new promising therapeutic tool to be considered in the future. In addition, since NOSC is considered an anti-tumoral safe drug, both in vitro and in vivo studies on neoplasic cells that are resistant to conventional anti-neoplasic drugs, shows the safety of this drug without severe side effects [39,74] and with a favorable pharmacokinetic profile [37]. This can be an advantage to adapt the use of NOSC to treat fibrotic conditions in mare endometrosis. Nevertheless, further studies must be performed to determine the adequate NOSC concentration, and the action of NOSC on the inhibition of pro-fibrotic effects of neutrophil myeloperoxidase. Once in vitro studies must not be directly extrapolated to in vivo organisms, in vivo studies in the mare must confirm our in vitro data.

## Figures and Tables

**Table 1:** Description of primer sequences for quantitative real-time polymerase chain reaction (qPCR).

Gene (Accession Number)	Sequence 5′-3′	Amplicon	References
*COL1A2* (XM_001492939.3)	Forward: CAAGGGCATTAGGGGACACA	196	[13,15,53]
	Reverse: ACCCACACTTCCATCGCTTC		
*RPL32* (XM_001492042.6)	Forward: AGCCATCTACTCGGCGTCA	144	[13,15,54]
	Reverse: GTCAATGCCTCTGGGTTTCC		

*RPL32*—ribosomal protein L32, *COL1A2*—collagen type I alpha 2 chain.

**Table 2:** Lactate dehydrogenase (LDH) activity of equine endometrial explants after 1, 24, or 48 h incubation. Results are presented as mean ± SEM. Different superscript letters indicate statistical differences within time of incubation (a, b: *p* < 0.05).

Time of Incubation	LDH Activity (%)
1 h	94.33 ± 0.91 ^a^
24 h	89.96 ± 0.73 ^b^
48 h	87.88 ± 0.85 ^b^

**Table 3:** Levels of significance (*p* values) for two- and three-way interactions between estrous cycle phases, treatment time, and cathepsin G (CAT) or noscapine (NOSC) treatments in the analyses of relative transcript *COL1A2* gene and COL1 protein relative abundance. The results were considered significant at *p* < 0.05.

	Evaluated Variable	
Isolated Factor/Interaction between Factors	*COL1A2* Gene	COL1 Protein
CAT	0.0003	0.0094
NOSC	<0.0001	0.4065
Time of treatment	0.4375	0.6498
Estrous cycle phase	0.8009	0.0013
CAT × NOSC	0.0028	0.0794
CAT × time of treatment	0.0707	0.0011
CAT × estrous cycle phase	0.8579	0.001
NOSC × time of treatment	0.8591	0.9252
NOSC × estrous cycle phase	0.1535	0.2856
Time of treatment × estrous cycle phase	0.0281	0.0359
CAT × NOSC × time of treatment	0.3104	0.7706
CAT × NOSC × estrous cycle phase	0.0089	0.3501
CAT × time of treatment × estrous cycle phase	0.0817	0.0126
NOSC × time of treatment × estrous cycle phase	0.9812	0.297

## Data Availability

Data will be available upon request to the corresponding author.

## Supplementary Materials

The following are available online at https://www.mdpi.com/article/10.3390/life11101107/s1, Table S1: List of differences found between noscapine (NOSC; 45 µg/mL) treatment, and cathepsin G (CAT; 0.1 and 1 µg/mL) for *COL1A2* transcription in equine endometrial explants, regardless of estrous cycle phase and treatment time. Table S2: List of differences found between noscapine (NOSC; 45 µg/mL) treatment, and the other performed treatments: (i) cathepsin G (CAT; 0.1 and 1 µg/mL) or (ii) CAT (0.1 and 1 µg/mL) + NOSC (45 µg/mL) for *COL1A2* transcription and COL1 protein relative abundance in equine endometrial explants from follicular (FP) or mid-luteal (MLP) phases, treated for 24 h or 48 h. Table S3: List of differences found in the same treatments between 24 h or 48 h of treatment, within each estrous cycle phase. Table S4: List of differences found in the same treatments between the follicular phase (FP) and mid-luteal phase (MLP) of the estrous cycle, within each treatment time. Table S5: Means and SEM for the performed treatments: (i) cathepsin G (CAT; 0.1 and 1 µg/mL); noscapine (NOSC; 45 µg/mL) or (ii) CAT (0.1 and 1 µg/mL) + NOSC (45 µg/mL) for *COL1A2* transcription and COL1 protein relative abundance in equine endometrial explants from follicular (FP) or mid-luteal (MLP) phases treated for 24 h or 48 h. Table S6: Levels of significance (*p* values) between cathepsin G (CAT) or noscapine (NOSC) treatments of equine endometrial explants from follicular phase (FP) or mid-luteal phase (MLP) treated for 24 h or 48 h in the analyses of relative transcript *COL1A2* gene. Table S7: Levels of significance (*p* values) between cathepsin G (CAT) or noscapine (NOSC) treatments of equine endometrial explants from follicular phase (FP) or mid-luteal phase (MLP) treated for 24 h or 48 h in the analyses of COL1 protein relative abundance. Table S8: Means and SEM for the performed treatments: (i) cathepsin G (CAT; 0.1 and 1 µg/mL); noscapine (NOSC; 45 µg/mL) or (ii) CAT (0.1 and 1 µg/mL) + NOSC (45 µg/mL) for *COL1A2* transcription and COL1 protein relative abundance in equine endometrial explants treated for 24 h or 48 h, independently of estrous cycle phase. Table S9: Levels of significance (*p* values) between cathepsin G (CAT) or noscapine (NOSC) treatments of equine endometrial explants treated for 24 h or 48 h in the analyses of relative transcript *COL1A2* gene. Table S10: Levels of significance (*p* values) between cathepsin G (CAT) or noscapine (NOSC) treatments of equine endometrial explants treated for 24 h or 48 h in the analyses of COL1 protein relative abundance. Table S11: Means and SEM for the performed treatments: (i) cathepsin G (CAT; 0.1 and 1 µg/mL); noscapine (NOSC; 45 µg/mL) or (ii) CAT (0.1 and 1 µg/mL) + NOSC (45 µg/mL) for *COL1A2* transcription and COL1 protein relative abundance in equine endometrial explants from follicular phase (FP) o mid-luteal phase (MLP), independently of time of treatment. Table S12: Levels of significance (*p* values) between cathepsin G (CAT) or noscapine (NOSC) treatments of equine endometrial explants from follicular phase (FP) or mid-luteal phase (MLP) in the analyses of relative transcript *COL1A2* gene. Table S13: Levels of significance (*p* values) between cathepsin G (CAT) or noscapine (NOSC) treatments of equine endometrial explants treated from follicular phase (FP) or mid-luteal phase (MLP) in the analyses of COL1 protein relative abundance.
