# Supplementary material for: The Inhibitory Effect of Noscapine on the In Vitro Cathepsin G-Induced Collagen Expression in Equine Endometrium
Source: Life (Basel). 2021 Oct 19;11(10):1107. doi: 10.3390/life11101107 (PMC8539599; doi:10.3390/life11101107)
Supplement: Supplementary file 1 [file life-11-01107-s001.zip › life-1405545-supplementary.pdf]

life

MDPI

Supplementary Materials

The Inhibitory Effect of Noscapine on the in Vitro Cathepsin G-Induced Collagen Expression in Equine Endometrium

Ana Amaral[1], Carina Fernandes[1], Anna Szóstek-Mioduchowska[2], Karolina Lukasik[2], Maria Rosa Rebordão[1,3], Pedro Pinto-Bravo[3], Dariusz Jan Skarzynski[2] and Graça Ferreira-Dias[1,*]

Table S1. List of differences found between noscapine (NOSC; 45 μg/mL) treatment, and cathepsin G (CAT; 0.1 and 1 μg/mL) for *COL1A2* transcription in equine endometrial explants, regardless of estrous cycle phase and treatment time.

Evaluated variable	Treatment comparison	P value	Figure
COL1A2 transcription	NOSC *vs* CAT 0.1	P < 0.01	1
	NOSC *vs* CAT 1	P < 0.001	

COL1A2—collagen type 1 α2; NOSC—noscapine; CAT 0.1—cathepsin G 0.1 μg/mL; CAT 1—cathepsin G 1 μg/mL.

Table S2. List of differences found between noscapine (NOSC; 45 μg/mL) treatment, and the other performed treatments: (i) cathepsin G (CAT; 0.1 and 1 μg/mL) or (ii) CAT (0.1 and 1 μg/mL) + NOSC (45 μg/mL) for *COL1A2* transcription and COL1 protein relative abundance in equine endometrial explants from follicular (FP) or mid-luteal (MLP) phases, treated for 24h or 48h.

Evaluated variable	Treatment comparison	P value	Figure
	NOSC *vs* CAT 0.1 FP 24 h	P < 0.01	2A
COL1A2 transcription	NOSC *vs* CAT 1 FP 24 h	P < 0.001	
	NOSC *vs* CAT 1 + NOSC FP 24 h	P < 0.01	
	NOSC *vs* CAT 1 MLP 24 h	P < 0.01	2B
	NOSC *vs* CAT 1 MLP 48 h	P < 0.01	
COL1 protein relative abundance	NOSC *vs* CAT 1 MLP 48 h	P < 0.001	2C
	NOSC *vs* CAT 1 + NOSC MLP 48 h	P < 0.001	

COL1A2—collagen type 1 α2; COL1—collagen type 1; NOSC—noscapine; CAT 0.1—cathepsin G 0.1 μg/mL; CAT 1—cathepsin G 1 μg/mL; FP—follicular phase; MLP—mid-luteal phase.

Table S3. List of differences found in the same treatments between 24h or 48h of treatment, within each estrous cycle phase**.**

Evaluated variables	Treatment comparison	P value	Figures
COL1A2 transcription	CAT 1 24 h FP *vs* CAT 1 48 h FP	P < 0.05	2A
	CAT 0.1 + NOSC 24 h FP *vs* CAT 0.1 + NOSC 48 h FP	P < 0.01	
COL1 protein relative abundance	CAT 0.1 24 h FP *vs* CAT 0.1 48 h FP	P < 0.01	2C
	CAT 1 24 h FP *vs* CAT 1 48 h FP	P < 0.05	
	CAT 1 + NOSC 24 h MLP *vs* CAT 1 + NOSC 48 h MLP	P < 0.05	2D

COL1A2—collagen type 1 α2; COL 1—collagen type I; CAT 0.1—cathepsin G 0.1 μg/mL; CAT 1—cathepsin G 1 μg/mL; NOSC—noscapine; FP—follicular phase; MLP—mid-luteal phase.

Table S4. List of differences found in the same treatments between the follicular phase (FP) and mid-luteal phase (MLP) of the estrous cycle, within each treatment time.

Evaluated variables	Treatment comparison	P value	Figures
COL1A2 transcription	CAT 1 48 h FP *vs* CAT 1 48 h MLP	P < 0.01	2A, 2B
	CAT 1 + NOSC 24 h FP *vs* CAT 1 + NOSC 24 h MLP	P < 0.01	
COL1 protein relative abundance	CAT 0.1 48 h FP *vs* CAT 0.1 48 h MLP	P < 0.05	2C, 2D
	CAT 1 + NOSC 48 h FP *vs* CAT 1 + NOSC 48 h MLP	P < 0.001	

COL1A2—collagen type 1 α2; COL 1—collagen type I; CAT 0.1—cathepsin G 0.1 µg/mL; CAT 1—cathepsin G 1 µg/mL; NOSC—noscapine; FP—follicular phase; MLP—mid-luteal phase.

Table S5. Means and SEM for the performed treatments: (i) cathepsin G (CAT; 0.1 and 1 µg/mL); noscapine (NOSC; 45 µg/mL) or (ii) CAT (0.1 and 1 µg/mL) + NOSC (45 µg/mL) for *COL1A2* transcription and COL1 protein relative abundance in equine endometrial explants from follicular (FP) or mid-luteal (MLP) phases treated for 24 h or 48 h.

		COL1A2		COL1	
**Time of treatment/ estrous cycle phase**	**Treatment**	Mean	SEM	Mean	SEM
24 h FP	CONTROL	93.7	20.5	99.5	9.1
	NOSC	68.0	20.9	111.2	11.1
	CAT 0.1	182.6	34.2	113.6	10.4
	CAT 0.1 + NOSC	36.3	16.5	110.7	12.1
	CAT 1	212.5	36.9	98.6	9.7
	CAT 1 + NOSC	213.3	43.8	100.2	11.5
48 h FP	CONTROL	92.0	20.3	99.5	9.1
	NOSC	79.1	26.7	131.0	14.7
	CAT 0.1	141.3	32.5	136.4	11.4
	CAT 0.1 + NOSC	53.9	22.0	100.9	11.6
	CAT 1	104.0	24.2	130.2	11.1
	CAT 1 + NOSC	58.4	22.9	105.3	13.2
24 h MLP	CONTROL	113.0	35.6	111.5	13.6
	NOSC	53.4	28.3	133.5	14.9
	CAT 0.1	103.9	34.2	154.8	18.5
	CAT 0.1 + NOSC	50.5	23.8	141.9	15.3
	CAT 1	250.3	53.0	129.6	14.7
	CAT 1 + NOSC	67.1	27.5	131.4	14.8
48 h MLP	CONTROL	77.7	20.9	89.1	8.6
	NOSC	102.2	27.7	101.7	11.6
	CAT 0.1	167.8	35.5	102.2	9.2
	CAT 0.1 + NOSC	52.4	24.3	114.0	15.9
	CAT 1	297.6	57.8	165.1	14.8
	CAT 1 + NOSC	66.1	27.3	190.2	17.8

COL1A2—collagen type 1 α2; COL1—collagen type 1; CAT 0.1—cathepsin G 0.1 µg/mL; CAT 1—cathepsin G 1 µg/mL; NOSC—noscapine 45 µg/mL; FP—follicular phase; MLP—mid-luteal phase.

Table S6. Levels of significance (P values) between cathepsin G (CAT) or noscapine (NOSC) treatments of equine endometrial explants from follicular phase (FP) or mid-luteal phase (MLP) treated for 24 h or 48 h in the analyses of relative transcript COL1A2 gene. The results were considered significant at P < 0.05.

	Control24hFP	Control 24h MLP	Control 48h FP	Control 48h MLP	NOSC 24h FP	NOSC 24h MLP	NOSC 48h FP	NOSC 48h MLP	CAT 0.1 24h FP	CAT 0.1 24h MLP	CAT 0.1 48h FP	CAT 0.1 48h MLP	CAT 0.1 + NOSC 24h FP	CAT 0.1 + NOSC 24h MLP	CAT 0.1 + NOSC 48h FP	CAT 0.1 + NOSC 48h MLP	CAT 1 24h FP	CAT 1 24h MLP	CAT 1 48h FP	CAT 1 48h MLP	CAT 1 + NOSC 24h FP	CAT 1 + NOSC 24h MLP	CAT 1 + NOSC 48h FP	CAT 1 + NOSC 48h MLP
Control24hFP		0.6297	0.9515	0.5842	0.3823	0.2789	0.6667	0.8037	0.0208	0.7948	0.2005	0.0585	0.0348	0.1923	0.201	0.2164	0.0034	0.0022	0.7427	0.0002	0.0077	0.4492	0.2634	0.4315
Control 24 h MLP	0.6297		0.5975	0.3742	0.2542	0.1921	0.4376	0.8078	0.1681	0.8524	0.5584	0.2798	0.0335	0.1359	0.1426	0.1514	0.0601	0.0288	0.8323	0.0056	0.0765	0.3006	0.1816	0.2892
Control 48 h FP	0.9515	0.5975		0.6241	0.4129	0.2975	0.7031	0.7633	0.018	0.7596	0.1828	0.052	0.0394	0.2083	0.2188	0.2339	0.0028	0.0019	0.6999	0.0002	0.0067	0.4771	0.285	0.4588
Control 48 h MLP	0.5842	0.3742	0.6241		0.7404	0.5021	0.9667	0.4728	0.0072	0.4998	0.0894	0.0227	0.1213	0.4016	0.4375	0.4395	0.0011	0.0008	0.4067	<.0001	0.0027	0.7582	0.5344	0.7364
NOSC 24 h FP	0.3823	0.2542	0.4129	0.7404		0.6815	0.7388	0.3155	0.0036	0.3515	0.0511	0.0121	0.2302	0.5845	0.6428	0.6296	0.0005	0.0004	0.2581	<.0001	0.0014	0.9783	0.7547	0.9558
NOSC 24 h MLP	0.2789	0.1921	0.2975	0.5021	0.6815		0.5126	0.2347	0.0077	0.2569	0.0533	0.0176	0.5851	0.9378	0.9873	0.98	0.0019	0.0011	0.2002	0.0001	0.0032	0.7274	0.8908	0.7449
NOSC 48 h FP	0.6667	0.4376	0.7031	0.9667	0.7388	0.5126		0.5477	0.0191	0.5614	0.1394	0.0457	0.155	0.423	0.4609	0.4586	0.0042	0.0023	0.4925	0.0003	0.0075	0.7517	0.5501	0.7318
NOSC 48 h MLP	0.8037	0.8078	0.7633	0.4728	0.3155	0.2347	0.5477		0.0676	0.9685	0.3549	0.1399	0.035	0.1639	0.1716	0.1834	0.017	0.0087	0.9596	0.0011	0.027	0.3725	0.2216	0.3581
CAT 0.1 24 h FP	0.0208	0.1681	0.018	0.0072	0.0036	0.0077	0.0191	0.0676		0.1129	0.3798	0.7626	<.0001	0.0026	0.0019	0.0032	0.5493	0.2688	0.056	0.0747	0.5742	0.0118	0.0031	0.0109
CAT 0.1 24 h MLP	0.7948	0.8524	0.7596	0.4998	0.3515	0.2569	0.5614	0.9685	0.1129		0.4305	0.1996	0.0539	0.191	0.2031	0.211	0.0371	0.018	0.9976	0.0032	0.0496	0.3952	0.2536	0.3815
CAT 0.1 48 h FP	0.2005	0.5584	0.1828	0.0894	0.0511	0.0533	0.1394	0.3549	0.3798	0.4305		0.578	0.0027	0.0276	0.0256	0.0322	0.1474	0.0685	0.3481	0.0136	0.1781	0.0871	0.0367	0.0822
CAT 0.1 48 h MLP	0.0585	0.2798	0.052	0.0227	0.0121	0.0176	0.0457	0.1399	0.7626	0.1996	0.578		0.0004	0.0073	0.0061	0.0088	0.3808	0.1829	0.1267	0.0471	0.4122	0.0281	0.0093	0.0262
CAT 0.1 + NOSC 24 h FP	0.0348	0.0335	0.0394	0.1213	0.2302	0.5851	0.155	0.035	<.0001	0.0539	0.0027	0.0004		0.613	0.5114	0.5697	<.0001	<.0001	0.0215	<.0001	<.0001	0.3131	0.4223	0.3263
CAT 0.1 + NOSC 24 h MLP	0.1923	0.1359	0.2083	0.4016	0.5845	0.9378	0.423	0.1639	0.0026	0.191	0.0276	0.0073	0.613		0.9154	0.9543	0.0005	0.0003	0.1308	<.0001	0.001	0.645	0.8116	0.663
CAT 0.1 + NOSC 48 h FP	0.201	0.1426	0.2188	0.4375	0.6428	0.9873	0.4609	0.1716	0.0019	0.2031	0.0256	0.0061	0.5114	0.9154		0.9634	0.0003	0.0002	0.134	<.0001	0.0008	0.7043	0.8885	0.724
CAT 0.1 + NOSC 48 h MLP	0.2164	0.1514	0.2339	0.4395	0.6296	0.98	0.4586	0.1834	0.0032	0.211	0.0322	0.0088	0.5697	0.9543	0.9634		0.0006	0.0004	0.1483	<.0001	0.0013	0.6866	0.8587	0.705
CAT 1 24 h FP	0.0034	0.0601	0.0028	0.0011	0.0005	0.0019	0.0042	0.017	0.5493	0.0371	0.1474	0.3808	<.0001	0.0005	0.0003	0.0006		0.5502	0.0121	0.2008	0.9879	0.0027	0.0005	0.0024
CAT 1 24 h MLP	0.0022	0.0288	0.0019	0.0008	0.0004	0.0011	0.0023	0.0087	0.2688	0.018	0.0685	0.1829	<.0001	0.0003	0.0002	0.0004	0.5502		0.0066	0.5434	0.5857	0.0015	0.0004	0.0014
CAT 1 48 h FP	0.7427	0.8323	0.6999	0.4067	0.2581	0.2002	0.4925	0.9596	0.056	0.9976	0.3481	0.1267	0.0215	0.1308	0.134	0.1483	0.0121	0.0066		0.0007	0.0215	0.325	0.1785	0.3111

CAT 1 48 h MLP	0.0002	0.0056	0.0002	<.0001	<.0001	0.0001	0.0003	0.0011	0.0747	0.0032	0.0136	0.0471	<.0001	<.0001	<.0001	<.0001	0.2008	0.5434	0.0007		0.2369	0.0002	<.0001	0.0002
CAT 1 + NOSC 24 h FP	0.0077	0.0765	0.0067	0.0027	0.0014	0.0032	0.0075	0.027	0.5742	0.0496	0.1781	0.4122	<.0001	0.001	0.0008	0.0013	0.9879	0.5857	0.0215	0.2369		0.0047	0.0012	0.0044
CAT 1 + NOSC 24 h MLP	0.4492	0.3006	0.4771	0.7582	0.9783	0.7274	0.7517	0.3725	0.0118	0.3952	0.0871	0.0281	0.3131	0.645	0.7043	0.6866	0.0027	0.0015	0.325	0.0002	0.0047		0.8046	0.98
CAT 1 + NOSC 48 h FP	0.2634	0.1816	0.285	0.5344	0.7547	0.8908	0.5501	0.2216	0.0031	0.2536	0.0367	0.0093	0.4223	0.8116	0.8885	0.8587	0.0005	0.0004	0.1785	<.0001	0.0012	0.8046		0.825
CAT 1 + NOSC 48 h MLP	0.4315	0.2892	0.4588	0.7364	0.9558	0.7449	0.7318	0.3581	0.0109	0.3815	0.0822	0.0262	0.3263	0.663	0.724	0.705	0.0024	0.0014	0.3111	0.0002	0.0044	0.98	0.825	

COL1A2—collagen type 1 α2; CAT 0.1—cathepsin G 0.1 μg/mL; CAT 1—cathepsin G 1 μg/mL; NOSC—noscapine 45 μg/mL; FP—follicular phase; MLP—mid-luteal phase.

Table S7. Levels of significance (P values) between cathepsin G (CAT) or noscapine (NOSC) treatments of equine endometrial explants from follicular phase (FP) or mid-luteal phase (MLP) treated for 24 h or 48 h in the analyses of COL1 protein relative abundance. The results were considered significant at P < 0.05.

	Control 24h FP	Control 24h MLP	Control 48h FP	Control 48h MLP	NOSC 24h FP	NOSC 24h MLP	NOSC 48h FP	NOSC 48h MLP	CAT 0.1 24h FP	CAT 0.1 24h MLP	CAT 0.1 48h FP	CAT 0.1 48h MLP	CAT 0.1 + NOSC 24h FP	CAT 0.1 + NOSC 24h MLP	CAT 0.1 + NOSC 48h FP	CAT 0.1 + NOSC 48h MLP	CAT 1 24h FP	CAT 1 24h MLP	CAT 1 48h FP	CAT 1 48h MLP	CAT 1 + NOSC 24h FP	CAT 1 + NOSC 24h MLP	CAT 1 + NOSC 48h FP	CAT 1 + NOSC 48h MLP
Control 24 h FP		0.458	0.9999	0.4017	0.4121	0.0459	0.0627	0.8811	0.3026	0.0051	0.0111	0.8385	0.4561	0.0147	0.9264	0.4179	0.944	0.0737	0.032	0.0001	0.9648	0.0592	0.7179	<.0001
Control 24 h MLP	0.458		0.4579	0.155	0.9858	0.2718	0.3275	0.5817	0.8987	0.0552	0.1623	0.5644	0.9647	0.1358	0.5489	0.9019	0.4336	0.36	0.2888	0.0085	0.522	0.3165	0.741	0.0005
Control 48 h FP	0.9999	0.4579		0.4018	0.4121	0.0459	0.0627	0.8811	0.3026	0.0051	0.0111	0.8385	0.4561	0.0147	0.9264	0.4179	0.944	0.0737	0.032	0.0001	0.9648	0.0592	0.7179	<.0001
Control 48 h MLP	0.4017	0.155	0.4018		0.112	0.0079	0.0116	0.3764	0.0672	0.0007	0.001	0.2979	0.1402	0.002	0.4079	0.1542	0.4592	0.0142	0.0035	<.0001	0.4356	0.0108	0.2963	<.0001
NOSC 24 h FP	0.4121	0.9858	0.4121	0.112		0.2222	0.2758	0.5543	0.8698	0.0372	0.1112	0.5273	0.976	0.099	0.5189	0.8813	0.3891	0.3077	0.2245	0.0035	0.49	0.2651	0.7306	0.0001
NOSC 24 h MLP	0.0459	0.2718	0.0459	0.0079	0.2222		0.904	0.0889	0.2658	0.3617	0.8724	0.0666	0.2294	0.6912	0.0803	0.3706	0.0446	0.8533	0.8575	0.1317	0.0736	0.9219	0.1539	0.0145
NOSC 48 h FP	0.0627	0.3275	0.0627	0.0116	0.2758	0.904		0.1149	0.3282	0.3063	0.7667	0.0895	0.282	0.6047	0.1043	0.4329	0.0607	0.9488	0.9653	0.1026	0.096	0.982	0.1913	0.0105
NOSC 48 h MLP	0.8811	0.5817	0.8811	0.3764	0.5543	0.0889	0.1149		0.4432	0.0127	0.0347	0.9767	0.5909	0.0349	0.9589	0.5256	0.8355	0.1311	0.0789	0.0009	0.9243	0.1096	0.8393	<.0001
CAT 0.1 24 h FP	0.3026	0.8987	0.3026	0.0672	0.8698	0.2658	0.3282	0.4432		0.0449	0.1364	0.4036	0.8518	0.1199	0.411	0.9834	0.2865	0.3651	0.2735	0.0041	0.3849	0.3158	0.6171	0.0002
CAT 0.1 24 h MLP	0.0051	0.0552	0.0051	0.0007	0.0372	0.3617	0.3063	0.0127	0.0449		0.3876	0.0079	0.0411	0.5853	0.0113	0.0925	0.0051	0.2792	0.2417	0.6627	0.0102	0.3162	0.0268	0.1688
CAT 0.1 48 h FP	0.0111	0.1623	0.0111	0.001	0.1112	0.8724	0.7667	0.0347	0.1364	0.3876		0.0187	0.1224	0.7737	0.0303	0.2563	0.0115	0.7121	0.69	0.1199	0.027	0.7862	0.0778	0.0095
CAT 0.1 48 h MLP	0.8385	0.5644	0.8385	0.2979	0.5273	0.0666	0.0895	0.9767	0.4036	0.0079	0.0187		0.5707	0.0226	0.9311	0.5092	0.7894	0.104	0.0508	0.0003	0.8929	0.0847	0.8454	<.0001
CAT 0.1 + NOSC 24 h FP	0.4561	0.9647	0.4561	0.1402	0.976	0.2294	0.282	0.5909	0.8518	0.0411	0.1224	0.5707		0.1064	0.5559	0.8654	0.431	0.3132	0.2359	0.0046	0.5272	0.2716	0.7609	0.0002
CAT 0.1 + NOSC 24 h MLP	0.0147	0.1358	0.0147	0.002	0.099	0.6912	0.6047	0.0349	0.1199	0.5853	0.7737	0.0226	0.1064		0.0311	0.2076	0.0147	0.5607	0.5305	0.2741	0.0281	0.6205	0.0694	0.0392
CAT 0.1 + NOSC 48 h FP	0.9264	0.5489	0.9264	0.4079	0.5189	0.0803	0.1043	0.9589	0.411	0.0113	0.0303	0.9311	0.5559	0.0311		0.497	0.8792	0.1193	0.07	0.0007	0.9653	0.0994	0.8015	<.0001
CAT 0.1 + NOSC 48 h MLP	0.4179	0.9019	0.4179	0.1542	0.8813	0.3706	0.4329	0.5256	0.9834	0.0925	0.2563	0.5092	0.8654	0.2076	0.497		0.3969	0.4684	0.4075	0.0214	0.4735	0.4208	0.6678	0.0018
CAT 1 24 h FP	0.944	0.4336	0.944	0.4592	0.3891	0.0446	0.0607	0.8355	0.2865	0.0051	0.0115	0.7894	0.431	0.0147	0.8792	0.3969		0.0711	0.0321	0.0002	0.9163	0.0574	0.6811	<.0001
CAT 1 24 h MLP	0.0737	0.36	0.0737	0.0142	0.3077	0.8533	0.9488	0.1311	0.3651	0.2792	0.7121	0.104	0.3132	0.5607	0.1193	0.4684	0.0711		0.9769	0.0894	0.11	0.9308	0.2138	0.0088
CAT 1 48 h FP	0.032	0.2888	0.032	0.0035	0.2245	0.8575	0.9653	0.0789	0.2735	0.2417	0.69	0.0508	0.2359	0.5305	0.07	0.4075	0.0321	0.9769		0.0559	0.0632	0.945	0.1528	0.0035

CAT 1 48 h MLP	0.0001	0.0085	0.0001	<.0001	0.0035	0.1317	0.1026	0.0009	0.0041	0.6627	0.1199	0.0003	0.0046	0.2741	0.0007	0.0214	0.0002	0.0894	0.0559		0.0006	0.1076	0.0031	0.2733
CAT 1 + NOSC 24 h FP	0.9648	0.522	0.9648	0.4356	0.49	0.0736	0.096	0.9243	0.3849	0.0102	0.027	0.8929	0.5272	0.0281	0.9653	0.4735	0.9163	0.11	0.0632	0.0006		0.0915	0.77	<.0001
CAT 1 + NOSC 24 h MLP	0.0592	0.3165	0.0592	0.0108	0.2651	0.9219	0.982	0.1096	0.3158	0.3162	0.7862	0.0847	0.2716	0.6205	0.0994	0.4208	0.0574	0.9308	0.945	0.1076	0.0915		0.1838	0.0111
CAT 1 + NOSC 48 h FP	0.7179	0.741	0.7179	0.2963	0.7306	0.1539	0.1913	0.8393	0.6171	0.0268	0.0778	0.8454	0.7609	0.0694	0.8015	0.6678	0.6811	0.2138	0.1528	0.0031	0.77	0.1838		0.0002
CAT 1 + NOSC 48 h MLP	<.0001	0.0005	<.0001	<.0001	0.0001	0.0145	0.0105	<.0001	0.0002	0.1688	0.0095	<.0001	0.0002	0.0392	<.0001	0.0018	<.0001	0.0088	0.0035	0.2733	<.0001	0.0111	0.0002	

COL1—collagen type 1; CAT 0.1—cathepsin G 0.1 μg/mL; CAT 1—cathepsin G 1 μg/mL; NOSC—noscapine 45 μg/mL; FP—follicular phase; MLP—mid-luteal phase.

Table S8. Means and SEM for the performed treatments: (i) cathepsin G (CAT; 0.1 and 1 μg/mL); noscapine (NOSC; 45 μg/mL) or (ii) CAT (0.1 and 1 μg/mL) + NOSC (45 μg/mL) for COL1A2 transcription and COL1 protein relative abundance in equine endometrial explants treated for 24h or 48h, independently of estrous cycle phase.

		COL1A2		COL1	
**Time of treatment/ estrous cycle phase**	**Treatment**	Mean	SEM	Mean	SEM
24 h	CONTROL	98.9	19.5	107.4	8.2
	NOSC	64.7	18.3	120.6	9.2
	CAT 0.1	144.9	25.2	131.4	10.1
	CAT 0.1 + NOSC	41.2	13.9	126.3	9.8
	CAT 1	229.9	31.8	112.9	8.6
	CAT 1 + NOSC	130.3	25.7	115.6	9.3
48 h	CONTROL	85.3	14.7	94.3	6.3
	NOSC	91.2	19.4	115.1	9.3
	CAT 0.1	154.2	24.1	118.5	7.3
	CAT 0.1 + NOSC	54.5	16.6	106.1	9.7
	CAT 1	189.6	28.1	147.6	9.2
	CAT 1 + NOSC	61.9	17.7	144.6	11.0

COL1A2—collagen type 1 α2; COL1—collagen type 1; CAT 0.1—cathepsin G 0.1 μg/mL; CAT 1—cathepsin G 1 μg/mL; NOSC—noscapine 45 μg/mL.

Table S9. Levels of significance (P values) between cathepsin G (CAT) or noscapine (NOSC) treatments of equine endometrial explants treated for 24h or 48h in the analyses of relative transcript COL1A2 gene. The results were considered significant at P < 0.05.

	Control 24h	Control 48h	NOSC 24h	NOSC 48h	CAT 0.1 24h	CAT 0.1 48h	CAT 0.1 + NOSC 24h	CAT 0.1 + NOSC 48h	CAT 1 24h	CAT 1 48h	CAT 1 + NOSC 24h	CAT 1 + NOSC 48h
Control 24h		0.5709	0.1955	0.7752	0.1415	0.0727	0.0166	0.0844	0.0003	0.0077	0.3233	0.1614
Control 48h	0.5709		0.3875	0.8093	0.0339	0.0118	0.0361	0.1764	<.0001	0.0006	0.1134	0.318
NOSC 24h	0.1955	0.3875		0.3226	0.0107	0.0038	0.2977	0.6786	<.0001	0.0002	0.0351	0.9139
NOSC 48h	0.7752	0.8093	0.3226		0.0879	0.0413	0.0353	0.1519	0.0002	0.0037	0.216	0.266
CAT 0.1 24h	0.1415	0.0339	0.0107	0.0879		0.7875	0.0002	0.0028	0.0347	0.2359	0.684	0.0071
CAT 0.1 48h	0.0727	0.0118	0.0038	0.0413	0.7875		<.0001	0.0009	0.0547	0.3353	0.4966	0.0025
CAT 0.1 + NOSC 24h	0.0166	0.0361	0.2977	0.0353	0.0002	<.0001		0.535	<.0001	<.0001	0.0016	0.3512
CAT 0.1 + NOSC 48h	0.0844	0.1764	0.6786	0.1519	0.0028	0.0009	0.535		<.0001	<.0001	0.0118	0.7572
CAT 1 24h	0.0003	<.0001	<.0001	0.0002	0.0347	0.0547	<.0001	<.0001		0.3339	0.0151	<.0001
CAT 1 48h	0.0077	0.0006	0.0002	0.0037	0.2359	0.3353	<.0001	<.0001	0.3339		0.1218	0.0001
CAT 1 + NOSC 24h	0.3233	0.1134	0.0351	0.216	0.684	0.4966	0.0016	0.0118	0.0151	0.1218		0.0262
CAT 1 + NOSC 48h	0.1614	0.318	0.9139	0.266	0.0071	0.0025	0.3512	0.7572	<.0001	0.0001	0.0262	

COL1A2—collagen type 1 α2; CAT 0.1—cathepsin G 0.1 μg/mL; CAT 1—cathepsin G 1 μg/mL; NOSC—noscapine 45 μg/mL.

Table S10. Levels of significance (P values) between cathepsin G (CAT) or noscapine (NOSC) treatments of equine endometrial explants treated for 24h or 48h in the analyses of COL1 protein relative abundance. The results were considered significant at P < 0.05.

	Control 24h	Control 48h	NOSC 24h	NOSC 48h	CAT 0.1 24h	CAT 0.1 48h	CAT 0.1 + NOSC 24h	CAT 0.1 + NOSC 48h	CAT 1 24h	CAT 1 48h	CAT 1 + NOSC 24h	CAT 1 + NOSC 48h
Control 24h		0.197	0.2731	0.5307	0.0603	0.3084	0.1346	0.9178	0.6388	0.0013	0.5041	0.0064
Control 48h	0.197		0.0162	0.0603	0.0016	0.0121	0.0052	0.2971	0.0766	<.0001	0.0542	<.0001
NOSC 24h	0.2731	0.0162		0.6667	0.4259	0.8534	0.6699	0.2751	0.5335	0.0389	0.6974	0.0922
NOSC 48h	0.5307	0.0603	0.6667		0.2308	0.7694	0.401	0.5032	0.8627	0.0141	0.9662	0.0399
CAT 0.1 24h	0.0603	0.0016	0.4259	0.2308		0.2932	0.7101	0.0732	0.1559	0.2388	0.2472	0.3751
CAT 0.1 48h	0.3084	0.0121	0.8534	0.7694	0.2932		0.5174	0.3084	0.6155	0.0132	0.8058	0.045
CAT 0.1 + NOSC 24h	0.1346	0.0052	0.6699	0.401	0.7101	0.5174		0.1404	0.2983	0.1133	0.4251	0.2103
CAT 0.1 + NOSC 48h	0.9178	0.2971	0.2751	0.5032	0.0732	0.3084	0.1404		0.5972	0.0026	0.4778	0.0091
CAT 1 24h	0.6388	0.0766	0.5335	0.8627	0.1559	0.6155	0.2983	0.5972		0.0062	0.8277	0.0223
CAT 1 48h	0.0013	<.0001	0.0389	0.0141	0.2388	0.0132	0.1133	0.0026	0.0062		0.0161	0.8356
CAT 1 + NOSC 24h	0.5041	0.0542	0.6974	0.9662	0.2472	0.8058	0.4251	0.4778	0.8277	0.0161		0.0439
CAT 1 + NOSC 48h	0.0064	<.0001	0.0922	0.0399	0.3751	0.045	0.2103	0.0091	0.0223	0.8356	0.0439	

COL1—collagen type 1; CAT 0.1—cathepsin G 0.1 μg/mL; CAT 1—cathepsin G 1 μg/mL; NOSC—noscapine 45 μg/mL.

Table S11. Means and SEM for the performed treatments: (i) cathepsin G (CAT; 0.1 and 1 μg/mL); noscapine (NOSC; 45 μg/mL) or (ii) CAT (0.1 and 1 μg/mL) + NOSC (45 μg/mL) for COL1A2 transcription and COL1 protein relative abundance in equine endometrial explants from follicular phase (FP) o mid-luteal phase (MLP), independently of time of treatment.

		COL1A2		COL1	
**Time of treatment/ estrous cycle phase**	**Treatment**	Mean	SEM	Mean	SEM
FP	CONTROL	92.8	14.5	99.5	6.5
	NOSC	74.8	17.0	119.5	9.1
	CAT 0.1	162.3	23.8	124.8	7.7
	CAT 0.1 + NOSC	43.9	13.5	105.7	8.4
	CAT 1	153.6	21.6	113.8	7.4
	CAT 1 + NOSC	123.7	23.7	103.0	8.8
MLP	CONTROL	91.2	19.5	101.9	7.9
	NOSC	79.9	21.0	116.2	9.4
	CAT 0.1	137.3	25.3	125.0	9.6
	CAT 0.1 + NOSC	51.5	17.1	126.7	11.1
	CAT 1	273.5	39.3	146.5	10.5
	CAT 1 + NOSC	66.6	19.4	159.4	11.6

COL1A2—collagen type 1 α2; COL1—collagen type 1; CAT 0.1—cathepsin G 0.1 μg/mL; CAT 1—cathepsin G 1 μg/mL; NOSC—noscapine 45 μg/mL; FP - folicular phase; MLP - mid-luteal phase.

Table S12. Levels of significance (P values) between cathepsin G (CAT) or noscapine (NOSC) treatments of equine endometrial explants from follicular phase (FP) or mid-luteal phase (MLP) in the analyses of relative transcript COL1A2 gene. The results were considered significant at $P < 0.05$.

	Control FP	Control MLP	NOSC FP	NOSC MLP	CAT 0.1 FP	CAT 0.1 MLP	CAT 0.1 + NOSC FP	CAT 0.1 + NOSC	CAT 1 FP	CAT 1 MLP	CAT 1 + NOSC FP	CAT 1 + NOSC
Control FP		0.9458	0.423	0.6139	0.0101	0.113	0.0178	0.0798	0.0169	<.0001	0.2509	0.2919
Control MLP	0.9458		0.5206	0.6881	0.0214	0.1422	0.0428	0.1282	0.0343	<.0001	0.283	0.3722
NOSC FP	0.423	0.5206		0.8511	0.0029	0.0364	0.1503	0.3355	0.0047	<.0001	0.0872	0.7489
NOSC MLP	0.6139	0.6881	0.8511		0.0119	0.0822	0.1348	0.2887	0.0184	<.0001	0.166	0.6397
CAT 0.1 FP	0.0101	0.0214	0.0029	0.0119		0.4716	<.0001	0.0003	0.7845	0.0125	0.2508	0.0028
CAT 0.1 MLP	0.113	0.1422	0.0364	0.0822	0.4716		0.0007	0.0051	0.6234	0.003	0.6928	0.0273
CAT 0.1 + NOSC FP	0.0178	0.0428	0.1503	0.1348	<.0001	0.0007		0.7234	<.0001	<.0001	0.0025	0.3233
CAT 0.1 + NOSC MLP	0.0798	0.1282	0.3355	0.2887	0.0003	0.0051	0.7234		0.0005	<.0001	0.0138	0.5544
CAT 1 FP	0.0169	0.0343	0.0047	0.0184	0.7845	0.6234	<.0001	0.0005		0.0053	0.3519	0.0044
CAT 1 MLP	<.0001	<.0001	<.0001	<.0001	0.0125	0.003	<.0001	<.0001	0.0053		0.0008	<.0001
CAT 1 + NOSC FP	0.2509	0.283	0.0872	0.166	0.2508	0.6928	0.0025	0.0138	0.3519	0.0008		0.0632
CAT 1 + NOSC MLP	0.2919	0.3722	0.7489	0.6397	0.0028	0.0273	0.3233	0.5544	0.0044	<.0001	0.0632	

COL1A2—collagen type 1 α2; CAT 0.1—cathepsin G 0.1 µg/mL; CAT 1—cathepsin G 1 µg/mL; NOSC—noscapine 45 µg/mL; FP—follicular phase; MLP—mid-luteal phase.

Table S13. Levels of significance (P values) between cathepsin G (CAT) or noscapine (NOSC) treatments of equine endometrial explants treated from follicular phase (FP) or mid-luteal phase (MLP) in the analyses of COL1 protein relative abundance. The results were considered significant at $P < 0.05$.

	Control FP	Control MLP	NOSC FP	NOSC MLP	CAT 0.1 FP	CAT 0.1 MLP	CAT 0.1 + NOSC FP	CAT 0.1 + NOSC MLP	CAT 1 FP	CAT 1 MLP	CAT 1 + NOSC FP	CAT 1 + NOSC MLP
Control FP		0.815	0.0709	0.1365	0.0124	0.0259	0.5561	0.0306	0.1427	0.0001	0.7475	<.0001
Control MLP	0.815		0.1416	0.2377	0.0405	0.0607	0.74	0.0649	0.2702	0.0008	0.925	<.0001
NOSC FP	0.0709	0.1416		0.8036	0.6542	0.6737	0.2652	0.6102	0.6288	0.0517	0.1926	0.0066
NOSC MLP	0.1365	0.2377	0.8036		0.4799	0.5108	0.4002	0.4662	0.84	0.0319	0.3009	0.0038
CAT 0.1 FP	0.0124	0.0405	0.6542	0.4799		0.9847	0.0971	0.885	0.3038	0.0927	0.066	0.0117
CAT 0.1 MLP	0.0259	0.0607	0.6737	0.5108	0.9847		0.1292	0.9075	0.3517	0.1297	0.0918	0.0219
CAT 0.1 + NOSC FP	0.5561	0.74	0.2652	0.4002	0.0971	0.1292		0.1275	0.4668	0.0026	0.8233	0.0002
CAT 0.1 + NOSC MLP	0.0306	0.0649	0.6102	0.4662	0.885	0.9075	0.1275		0.3272	0.196	0.0919	0.0421
CAT 1 FP	0.1427	0.2702	0.6288	0.84	0.3038	0.3517	0.4668	0.3272		0.0104	0.3462	0.0008
CAT 1 MLP	0.0001	0.0008	0.0517	0.0319	0.0927	0.1297	0.0026	0.196	0.0104		0.0017	0.4022
CAT 1 + NOSC FP	0.7475	0.925	0.1926	0.3009	0.066	0.0918	0.8233	0.0919	0.3462	0.0017		0.0001
CAT 1 + NOSC MLP	<.0001	<.0001	0.0066	0.0038	0.0117	0.0219	0.0002	0.0421	0.0008	0.4022	0.0001	

COL1—collagen type 1; CAT 0.1—cathepsin G 0.1 µg/mL; CAT 1—cathepsin G 1 µg/mL; NOSC—noscapine 45 µg/mL; FP—follicular phase; MLP—mid-luteal phase.
